# Susceptibility to Photosynthesis Suppression From Extreme Storms Is Highly Site‐Dependent

**DOI:** 10.1111/gcb.70257

**Published:** 2025-05-22

**Authors:** Erica L. McCormick, Caroline A. Famiglietti, Dapeng Feng, Anna M. Michalak, Alexandra G. Konings

**Affiliations:** ^1^ Department of Earth System Science Stanford University Stanford California USA; ^2^ Hydrosat Inc. Washington DC USA; ^3^ Stanford Institute for Human‐Centered Artificial Intelligence (HAI) Stanford University Stanford California USA; ^4^ Department of Global Ecology Carnegie Institution for Science Stanford California USA

**Keywords:** carbon uptake, climate extremes, ecohydrology, eddy covariance, extreme storms, machine learning, photosynthesis, waterlogging

## Abstract

Extreme storms are becoming more intense and frequent under climate change. Although these extreme wet events are smaller in extent and duration than drought events, recent evidence suggests the global impact of both extremes is similar. However, the impact of individual extreme storms on photosynthesis—and therefore on vegetation and the carbon cycle—remains difficult to predict, as photosynthesis may be suppressed via waterlogging or increased by the alleviation of moisture stress. Here, we use random forest models to calculate daily photosynthesis anomalies attributable to extreme soil moisture using data from 54 FLUXNET sites across the globe. We hypothesize that photosynthesis' response to a given extreme event is primarily controlled by storm intensity, and to a lesser degree by site vegetation, climate, soil, and topography. However, we find instead that photosynthesis responses are better explained by site characteristics (soil texture, climate, topography, and vegetation density) than by storm intensity, such that the likelihood of waterlogging from a given storm is heavily site‐dependent. Although storms that induce waterlogging are roughly as common as those that induce stress alleviation overall, photosynthesis rarely declines at sites not prone to waterlogging. Instead, photosynthesis anomalies at these sites show a much weaker relationship with storm intensity. Increasingly intense storms are therefore unlikely to impact all locations equally. This highlights the potential to use site characteristics to enhance prediction of storm effects on ecosystems and the land carbon sink.

## Graphical abstract

Extreme storms are becoming more intense and frequent under climate change. However, the impact of individual extreme storms on photosynthesis—and therefore on vegetation and the carbon cycle—remains difficult to predict. Here, we use random forest models to calculate daily photosynthesis anomalies attributable to extreme soil moisture using data from 54 FLUXNET sites across the globe. We find that photosynthesis responses are better explained by site characteristics than by storm intensity, such that the likelihood of waterlogging from a given storm is heavily site‐dependent. Increasingly intense storms under climate change are therefore unlikely to impact all locations equally.

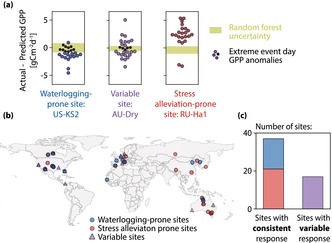

## 1 Introduction

Extreme climate events, such as droughts (Cook et al. 2022) and floods (Milly et al. 2002), are projected to become more frequent and intense with climate change. Wet extremes in particular are expected to increase in severity and frequency in tandem with rising air temperatures, which set the moisture holding capacity of the atmosphere (Donat et al. 2016; Trenberth 2011). However, in many regions, the intensity of short‐duration precipitation extremes has increased even faster than expected based on warming temperatures alone (Fowler et al. 2021; Westra et al. 2014).

The effects of drought on photosynthesis are far more commonly studied than those of wet extremes, perhaps in part because the larger spatial extent and greater average duration of drought are assumed to imply a greater impact. However, several recent lines of evidence suggest that the total impact of wet extremes may not be much smaller than that of drought. For example, negative extremes in annual tree ring widths across the globe are almost as likely to be caused by years with wet extremes as by years with drought (< 2% difference in occurrence), with similar results obtained when annual‐scale NDVI was considered (Yang et al. 2023), despite wet extremes likely occurring during only a small fraction of any given year. Furthermore, a global analysis by Famiglietti et al. (2021) found almost as many pixels where the integrated effect of wet anomalies on NDVI was greater than the integrated effect of drought as pixels where the opposite was true. In other words, it is as common for the total change in vegetation productivity induced by wet extremes to be larger than the total change induced by dry extremes at a given location than vice versa. Crop record and insurance claim analyses likewise reveal that magnitudes of maize yield reduction attributable to drought and waterlogging are similar across the United States (Li et al. 2019). Waterlogging in crops more generally has cost an estimated $19 billion USD over the last 50 years globally (Liu et al. 2023). Indeed, negative impacts from wet extremes are sufficiently common that even studies designed to interrogate vegetation response to drought have revealed negative vegetation responses to waterlogging (Fu et al. 2022; Stocker et al. 2018).

However, it remains impossible to predict photosynthesis response to an individual extreme wet event. In part, this is because extreme wet events do not always suppress photosynthesis. Although commonly associated with declines in gross primary productivity (GPP) and growth (e.g., Malik et al. 2002; Ohta et al. 2014; Terazawa et al. 1992), excess moisture can also enhance productivity by alleviating preexisting water stress (e.g., Heisler‐White et al. 2008; Li et al. 2019). Whether competing waterlogging or water stress alleviation processes will dominate the impact on vegetation for a given storm event depends on factors that vary from storm to storm (e.g., storm duration, seasonal timing, and inundating water chemistry; Black 1984; Froend et al. 1987; Kozlowski 1997) as well as with site characteristics such as vegetation type and phenology (Dat and Parent 2012; Kreyling, Beierkuhnlein, et al. 2008; Niinemets and Valladares 2006), soil and topographical drainage effects (Mattos et al. 2023), and climate (Li et al. 2019). However, the relative impact of these factors is unknown. For example, one might expect that increasing storm intensities will be the primary driver increasing the likelihood of waterlogging (causing negative effects on photosynthesis to dominate) because higher degrees of prolonged saturation are expected to induce correspondingly negative impacts on vegetation physiology. However, it is unclear whether this is true, that is, whether increasingly extreme storms really do increase waterlogging. Instead, site characteristics such as soil texture, topography, and vegetation type might mitigate or amplify the negative physiological impacts from soil saturation.

To address this gap, we use machine learning and in situ measurements of soil moisture and photosynthesis to estimate the impact of extreme soil wetness on daily GPP at 54 FLUXNET sites globally. We compare moisture‐driven anomalies in GPP across these sites and across a range of storms to explore how commonly extreme wet events cause reductions in photosynthesis. We further test our hypothesis that the amount of excess moisture—rather than characteristics of the impacted sites—controls whether photosynthesis will increase or decrease in response to extreme storms.

## 2 Methods

**FIGURE 1:**
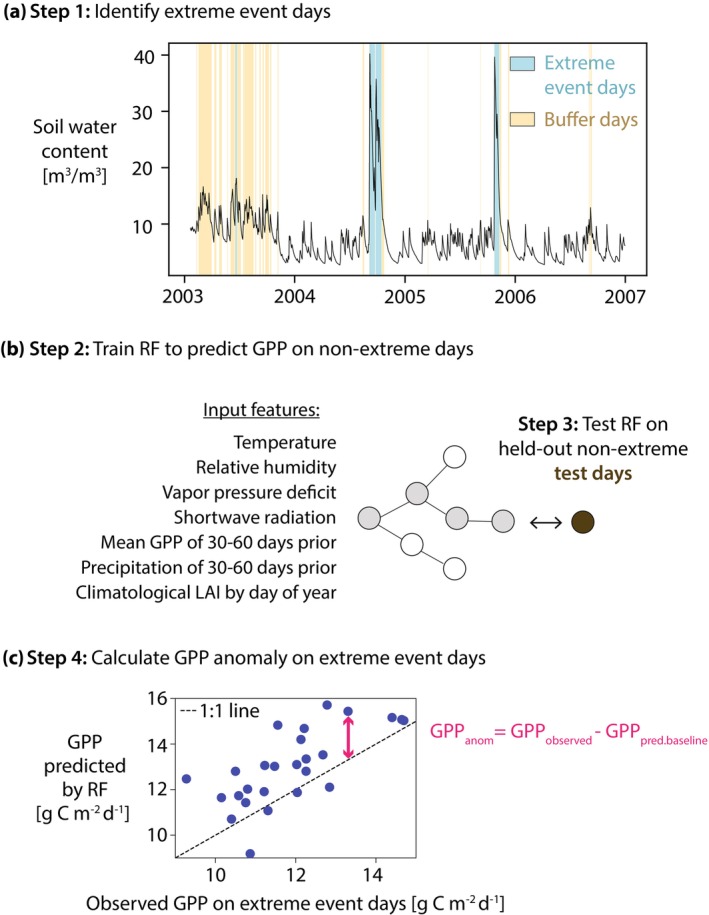
Overview of GPP anomaly calculation. (a) Daily soil moisture at a sample FLUXNET site (here, US‐KS2). We identify extreme wet event days (shown in blue) as half‐hourly soil moisture exceeding the 95th percentile for the site for at least 48 consecutive hours. “Buffer days,” shown in yellow, are removed from the random forest (RF) training dataset and are defined by soil moisture exceeding the 80th percentile for at least 24 consecutive hours. (b) A random forest model is built for each site to predict GPP on non‐extreme days. The feature inputs are temperature, relative humidity, vapor pressure deficit, incoming shortwave solar radiation, wind speed, mean day of year leaf area index (LAI), cumulative precipitation for the preceding 30–60 days, and mean GPP for the preceding 30–60 days. Note that soil moisture is not included as a model input. (c) The trained random forest models are used to predict GPP on extreme wet event days, where the difference between the observed and predicted GPP is the GPP anomaly (GPP_anom_). Blue dots show the observed versus predicted GPP for each extreme wet event day and the pink arrow highlights GPP_anom_ for 1 day.

We use random forest models to calculate the direction and magnitude of GPP change attributable to excess soil moisture at 54 FLUXNET2015 sites (Pastorello et al. 2020). For each site, we identify “extreme wet event days” where soil moisture levels are at extremely high values relative to the site's soil moisture distribution. To calculate the change in GPP attributable to extreme soil moisture on these extreme event days, we first train a random forest model for each site to predict GPP using only meteorological data from non‐extreme days. The trained model for each site is then used to generate GPP predictions for the extreme event days. The resulting GPP predictions serve as a “baseline” or expected GPP based on the conditions associated with each storm (such as wind speed and humidity), without reflecting changes in GPP induced specifically by extremely high soil moisture, which is not included as a model input. Comparing the random forest predicted GPP to the observed GPP allows calculation of daily GPP anomalies attributable to extreme soil moisture. This approach is outlined in Figure 1. We then use these anomalies to evaluate the frequency of waterlogging‐dominated versus water stress alleviation‐dominated vegetation response to wet extremes, comparing behavior during individual events as well as across sites.

### 2.1 Preparing the FLUXNET Data

We start with the 82 FLUXNET sites where both soil moisture and GPP are measured and where the dominant land cover is forest, shrubland, savanna, or grassland. These land cover categories are chosen in order to avoid nonvegetated land cover types. We consider only time periods when vegetation is likely to be photosynthetically active by analyzing only the consecutive range of days that have temperatures above 1°C for all years in the FLUXNET record. For sites that never freeze, we analyze data over the full year.

All FLUXNET half‐hourly measurements are aggregated to daily averages using only the daytime measurements, assumed to range from 6 am to 6 pm. We use only daytime data in order to maximize the number of days with high‐quality eddy covariance data by avoiding filtering whole days due to low‐quality data at night, when GPP is negligible. All variables (not just GPP) are aggregated over these same daytime hours because these are the meteorological conditions most closely corresponding to the times when photosynthesis occurs. If precipitation occurs during more than two of the daytime half‐hourly measurements, or if more than two half‐hourly quality‐control flags were neither “measured” nor “good‐quality gapfill,” we exclude the day from further analysis in order to avoid conditions with poor‐quality eddy covariance data. For simplicity, we only use GPP derived from the daytime partitioning method used to process the eddy covariance observations, although GPP derived from nighttime partitioning is also considered for sensitivity analyses.

“Extreme wet event days” at each site are defined as those when the shallowest reported depth of soil moisture exceeds the 95th percentile half‐hourly value for a given site for at least 48 consecutive hours. We define extreme event days in terms of extremely high soil moisture, rather than precipitation, because the eddy covariance quality filtering excludes many days with active precipitation. Therefore, the extreme event days investigated here generally occur shortly after a precipitation event has concluded.

For creating the non‐extreme model training and evaluation dataset, we also exclude a buffer of days surrounding the extreme event days. This is because we expect the degree of wetness necessary to induce a potential decline in GPP to vary between sites, but we do not know this amount a priori. Without a buffer window, selecting a specific soil moisture threshold that is too high would allow “leakage” of extreme conditions into the training data. However, selecting a specific soil moisture threshold that is too low would lead the analysis to cover all wet events, rather than only extremes. Using a 95th percentile soil moisture threshold for extreme wetness in combination with a buffer window avoids both potential pitfalls. We therefore exclude from training all periods when half‐hourly soil moisture exceeds the 80th percentile for the site for at least 24 consecutive hours. That is, training is performed when recent soil moisture is below its local 80th percentile, the buffer window covers the 80th to 95th percentile, and days with soil moisture above the 95th percentile are considered extreme wet event days.

### 2.2 Calculating the Change in GPP Attributable to Extreme Soil Moisture

We first predict GPP under non‐extreme conditions using a random forest model trained for each site using input features chosen to capture the meteorological drivers, seasonality, and interannual variability of GPP. These input features are: temperature, vapor pressure deficit, relative humidity, wind speed, and shortwave incoming radiation (all as a daily average), the cumulative precipitation and mean GPP of 30–60 days before the measurement day, and climatological leaf area index. All of the data for the input features are provided from FLUXNET with the exception of daily leaf area index from MODIS (MOD15A2H version 6.1; Myneni et al. 2021), from which we calculate a mean day of year value using the years 2000–2023. In each case, LAI for the 500‐m pixel in which the FLUXNET site occurs is used. The features representing recent precipitation and GPP and the climatological leaf area index are included so that GPP seasonality and interannual variability are captured in the predictions without including information about moisture or photosynthesis conditions on the specific day of the prediction. Note that all information about soil moisture or precipitation on the day of the prediction must be excluded from the models so that our predictions reflect “baseline” GPP in the absence of extreme soil moisture.

Prior to training the random forest models, both the input features and the target (in this case, GPP) are transformed by a min–max scaler. The hyperparameters are tuned via randomized grid search using 10‐fold double‐nested cross‐validation. We additionally withhold 25% of the non‐extreme days from the random forest training dataset to perform model evaluation. The model performance is calculated for each site by comparing the predicted GPP to the observed GPP for this withheld validation dataset. We remove all sites with a model performance of *R* ^2^ < 0.7 from further analysis. All random forest modeling was performed with the scikit‐learn package (version 1.2.2) in Python (Pedregosa et al. 2011).

After training and validating the random forest model to predict GPP on non‐extreme days for each site, we use the models to predict GPP on the extreme event days. The GPP anomaly (GPP_anom_) attributable to extreme soil moisture is calculated as the observed GPP on the wet extreme days minus the random forest predicted “baseline” GPP (GPP_pred.baseline_) on the same wet extreme day (see Figure 1c).

(1) $${\mathrm{GPP}}_{\text{anom}}={\mathrm{GPP}}_{\text{observed}}-{\mathrm{GPP}}_{\text{pred}.\;\text{baseline}}$$

Negative GPP_anom_ (observed less than predicted GPP) indicates vegetation waterlogging dominates over water stress alleviation, whereas positive GPP_anom_ can be interpreted as water stress alleviation dominating any potential waterlogging.

To reduce the impact of model uncertainty on our interpretation of GPP_anom_, we restrict our analysis to those days where GPP_anom_ falls outside of the random forest uncertainty, defined as the 25th to 75th percentile range of the withheld test day anomaly distribution for each site. We retain all sites that have at least 4 days with GPP_anom_ outside of this uncertainty.

### 2.3 Explaining the GPP Anomaly as a Function of Site Characteristics and Storm Conditions

When comparing GPP_anom_ across all sites, we first account for the spatial variability in productivity across sites by normalizing GPP_anom_ by the mean and standard deviation of the observed daily GPP at each site.

(2) $${\mathrm{GPP}}_{\text{anom},\mathrm{norm}}=\frac{{\mathrm{GPP}}_{\text{anom}}-\text{mean}\left({\mathrm{GPP}}_{\text{observed}}\right)}{\mathrm{std}\left({\mathrm{GPP}}_{\text{observed}}\right)}$$

Above, mean(GPP_observed_) and std(GPP_observed_) represent the time‐averaged mean and standard deviation for each site. The observed GPP used to calculate these site statistics is the same high‐quality, daytime average GPP values from the full FLUXNET record of each site and corresponds to the same data used for model training, testing, and wet extreme days.

To test the hypothesis that storm intensity controls vegetation response to extreme wet events, we try to explain GPP_anom,norm_ as a function of site and storm characteristics by using a second set of random forest models and examining the relative explanatory power of each input feature. We consider the site characteristics of soil texture, climate, topography, and vegetation density. For the storm characteristics, we consider soil moisture, storm intensity, and wind speed (see Table 1). There are many possible variables, which could be used to represent each of these characteristics. However, including multiple, highly cross‐correlated variables into a single random forest model would prevent us from robustly explaining which features are most important for explaining variability in the GPP anomaly. Therefore, we do not include all of the possible explanatory variables as feature inputs into a single random forest model. Instead, we choose just one variable to represent each site and storm characteristic, respectively, and we train one model for every combination of possible variables. For example, one model could represent the site characteristic of “soil texture” using just the variable of “soil porosity,” whereas a second model would instead represent soil texture using only “soil bulk density.” Table 1 shows the variables we considered for each characteristic. We train 144 models to exhaust all possible combinations of variables for each site and storm characteristic. We also train models using only storm or only site information, but not both. Model hyperparameters were selected for each of the full, storm, and site models individually via randomized grid search using double‐nested cross‐validation with threefold for hyperparameter tuning and fivefold for model validation. Allowing for the separate optimization of hyperparameters between model types ensures that models maximize their use of the information content of available observations, without imposing artificial performance restrictions due to differences in the number of input features or observations.

**TABLE 1:** Site and storm characteristics used to explain GPP_anom,norm_ across sites. Each model uses a different combination of variables so that each site and storm characteristic is represented.

		Data	Source
**Site characteristics**			
Soil texture	Saturated hydraulic conductivity (0–5 cm)	SoilGrids	Poggio et al. (2021)
	Bulk density (0–5 cm)	SoilGrids	Poggio et al. (2021)
	Porosity (0–5 cm)	SoilGrids	Poggio et al. (2021)
	Skewness of soil moisture distribution across site record	FLUXNET2015	Pastorello et al. (2020)
Climate	Mean annual precipitation	FLUXNET2015	Pastorello et al. (2020)
	Mean annual temperature	FLUXNET2015	Pastorello et al. (2020)
	Aridity index (P/PET)	MODIS FLUXNET2015	Running et al. (2021), Pastorello et al. (2020)
Topography	Elevation	MERIT Hydro	Yamazaki et al. (2019)
	Height above nearest drainage (HAND)	MERIT Hydro	Yamazaki et al. (2019)
	Topographic wetness index (TWI)	MERIT Hydro	Yamazaki et al. (2019)
Vegetation	Mean site leaf area index	MODIS	Myneni et al. (2021)
**Storm characteristics**			
Soil moisture	Daily soil moisture	FLUXNET2015	Pastorello et al. (2020)
	Pre‐storm (antecedent) soil moisture	FLUXNET2015	Pastorello et al. (2020)
	Maximum storm soil moisture up to day of measurement	FLUXNET2015	Pastorello et al. (2020)
Storm intensity	Storm duration up to day of measurement	FLUXNET2015	Pastorello et al. (2020)
	Cumulative storm precipitation amount up to day of measurement	FLUXNET2015	Pastorello et al. (2020)
Wind speed	Maximum storm wind speed up to day of measurement	FLUXNET2015	Pastorello et al. (2020)

The soil texture characteristics are all calculated from the SoilGrids (Poggio et al. 2021) dataset. We use soil porosity, bulk density, clay fraction, and sand fraction provided for the upper 5 cm of soil, and we calculate saturated hydraulic conductivity after Cosby et al. (1984) as a function of sand and clay fraction. For the climate at each site, we calculate the mean annual precipitation and temperature from the full FLUXNET record at each site. We calculate the aridity index by dividing the mean annual precipitation by the cumulative mean annual potential evapotranspiration (PET) over the years 2001–2023 from MODIS (MOD16A2 version 6; Running et al. 2021). All topography data are from the MERIT Hydro (version 1.0.1; Yamazaki et al. 2019) dataset. Elevation and height above nearest drainage (HAND) are provided directly, and we use the provided slope angle and upslope drainage area to calculate the topographic wetness index (TWI). HAND is calculated as the difference between elevation and the elevation of the nearest drainage based on numerically estimated flow paths from a digital elevation model (Nobre et al. 2011). TWI is calculated as the natural logarithm of the ratio between the upslope drainage area and the tangent of the slope angle (Beven and Kirkby 1979). Both HAND and TWI can be used to capture the effect of topography on water drainage and availability for normalized comparisons of sites across space. Vegetation density is represented by a single variable in each model, which is the mean site leaf area index (Myneni et al. 2021) over the years 2000–2023.

For the storm metrics, we define storms as back‐to‐back days with precipitation and associate each extreme event day with the nearest preceding or ongoing storm. All data used for calculating the storm metrics come from the daily FLUXNET data. The storm characteristic of soil moisture is represented either by the observed soil moisture on the day of the measurement, the antecedent soil moisture before the storm, or the maximum storm soil moisture up to the day of the measurement. The storm intensity is represented either by the variable of storm amount (calculated as the total precipitation amount across the storm, i.e., across all consecutive days with rainfall) or by the storm length, both calculated up to the day of the measurement. Finally, wind speed is represented by the storm maximum wind speed up to the day of the measurement. All of the storm characteristics represent maximum conditions so far (i.e., up to the measurement day), even if the storm is ongoing.

### 2.4 Characterizing Site‐Specific Variability in Whether an Extreme Event Induces Waterlogging or Water Stress Alleviation

To explore the drivers of waterlogging versus water stress alleviation across all sites and extreme event days, we classify sites as either waterlogging‐prone, stress alleviation‐prone, or variable by comparing the mean of the GPP_anom_ at each site to the 25th to 75th percentile confidence interval of the random forest uncertainty at that site. If the mean of the GPP_anom_ falls within the random forest uncertainty, we label those sites as having a “variable” response. Otherwise, waterlogging‐prone sites are those where the mean of the GPP_anom_ is below the 25th percentile of the random forest confidence interval and stress alleviation‐prone sites are those where the mean of GPP_anom_ exceeds the 75th percentile of the confidence interval. Because the mean of the confidence interval is zero, waterlogging‐prone sites are those where the mean of GPP_anom_ is negative and stress alleviation‐prone sites are those where the mean of GPP_anom_ is positive.

Two sites were manually reclassified based on visual inspection (Figure S6). US‐Wkg was manually reclassified from waterlogging‐prone to variable and US‐Var was reclassified manually from stress alleviation‐prone to variable. These sites have long data records and high random forest performance, which results in very narrow random forest uncertainty. Therefore, the mean of GPP_anom_ was outside of the random forest confidence interval despite the site having a variable response to extreme events. The main results are qualitatively unchanged with or without this manual reclassification.

We compare these site categories to the species‐specific sensitivity to waterlogging to determine if the species cover at each of the sites affects their response to extreme wet events. Specifically, we use the TRY database of plant traits (Kattge et al. 2011) to compare the dominant species “tolerance to waterlogging” trait where that trait information is available for species listed as dominant at a site in FLUXNET. This trait is measured on a scale from 1 to 5 (Whitlow and Harris 1979) where 1 is “very intolerant (does not tolerate water‐saturated soils for more than a few days during the growing season),” 2 is “intolerant (tolerates 1–2 weeks of waterlogging during the growing season),” and extends to 5, very tolerant, where vegetation “survives deep, prolonged waterlogging for more than one year.” Waterlogging tolerance is typically measured by transects overlapping inundated areas and vegetation mortality is assessed and compared with waterlogging duration. Although the “tolerance to waterlogging” trait is not very granular (given that it has only five possible values), it is the only relevant trait in TRY.

We also compare the site categories to the simulated depth to water table from Fan et al. (2013), which combines in situ estimates of groundwater depth with a model forced by climate and topography to simulate a global best estimate at 1‐km resolution.

## 3 Results

### 3.1 Calculating GPP Anomalies Attributable to Extreme Wet Events

Across FLUXNET2015, 82 sites meet our analysis criteria. The random forest performance (*R* ^2^) for predicting GPP during non‐extreme conditions exceeds 0.7 for 62 of these sites. We additionally filter eight sites with fewer than four extreme event days, leaving 54 sites for analysis. See Table S1 for details on all sites, which are concentrated in North America, Europe, Asia, and Australia. Across these sites, we identify 1443 extreme event days with high‐quality daytime eddy covariance data. After calculating the GPP anomaly (GPP_anom_) for each of these extreme event days, we retain the 1072 days (74%) where GPP_anom_ falls outside of the random forest uncertainty. We also associate each extreme event day with a preceding precipitation event or “storm.” Across all sites, 87% of storms have a duration of 1 week or less and 56% last 3 days or less (Figure S1).

The distribution and cross‐correlations of the input features to the random forest models are similar across the non‐extreme days (used for model training and testing) and the extreme wet event days (when predictions are made) (Figure S2). This suggests our predictions of “baseline” or expected GPP in the absence of extreme soil moisture are not influenced by the extreme wetness via meteorological cross‐correlation. That the range of values for each feature are similar between the testing and prediction datasets also builds confidence that the random forest models are not extrapolating in order to predict GPP on extreme wet event days (Figure S3). This makes sense because we have intentionally excluded input features related to soil moisture, which is the variable that diverges dramatically between the non‐extreme and extreme days. Partial dependence plots across all input features are smooth (Figure S4), further building confidence that the modeled relationships are physically meaningful. Although we present the main results using GPP derived from the daytime partitioning method, our findings are qualitatively similar if using nighttime‐partitioned GPP (Figure S5).

### 3.2 Site Characteristics, Rather Than Storm Conditions, Better Explain Photosynthesis Response to Extreme Wet Events

**FIGURE 2:**
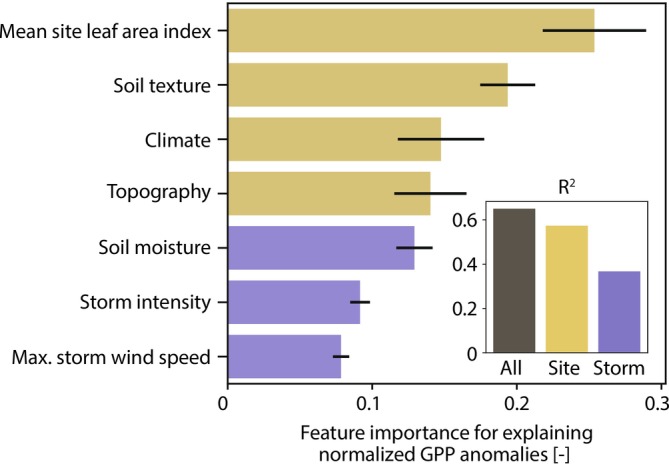
The permutation feature importance for explaining the normalized GPP anomaly (GPP_anom,norm_) across all sites as a function of site characteristics and storm characteristics. Bars and black lines show the mean and standard deviation of the feature importance for each input feature across the 10 models with the highest prediction performance. Features representing site characteristics are shown in yellow and those representing storm characteristics are shown in purple. Note that each model uses just one variable to represent each site and storm characteristic, respectively. See Table 1 for the full list of possible variables representing each characteristic. Inset shows the performance of the best model using all characteristics (black), just site characteristics (*R* ^2^ = 0.57, yellow), or just storm characteristics (*R* ^2^ = 0.37, purple).

The top 10 best‐performing models built to explain the normalized GPP anomalies (GPP_anom,norm_) across all sites and all extreme event days achieved an *R* ^2^ of 0.65 (*±*0.002 standard deviation). Contrary to our initial hypothesis that storm conditions would be the primary driver of GPP response, we find instead that site characteristics consistently have greater feature importance than storm conditions (Figure 2). Across every model—regardless of feature or hyperparameter choice—the variables representing vegetation density and soil texture have the first and second largest feature importance, respectively. Furthermore, each site characteristic consistently has a higher feature importance than any storm characteristic. This greater importance of site characteristics than storm conditions is also highlighted by the fact that a random forest model trained to predict GPP_anom,norm_ using only site characteristics achieved an *R* ^2^ of 0.57 (*±*0.003) (only 13% below that of the full model's *R* ^2^ of 0.65). In contrast, a model built using only storm conditions achieved an *R* ^2^ of only 0.37 (*±*0.03). The minimal improvement in model performance gained by including storm conditions over and above site characteristics alone (*R* ^2^ = 0.65 vs. *R* ^2^ = 0.57) compared to the performance using storm conditions alone (*R* ^2^ = 0.37) suggests that the information relevant to predicting GPP_anom,norm_ provided by storm conditions overlaps with that provided by site characteristics via cross‐correlation.

### 3.3 The Majority of Sites Are Either Waterlogging‐Prone or Stress Alleviation‐Prone

To further understand the overall behavior of the GPP anomalies, we examine their sign. Across all sites, 49% (528) of the 1072 extreme wet event days outside of the random forest uncertainty show a waterlogging‐dominated response (negative GPP_anom_). Because these anomalies are calculated at FLUXNET sites that are not necessarily representative of the broader land surface, this balance of waterlogging and stress alleviation is not necessarily true globally. Nevertheless, that around half of all extreme event days are associated with a waterlogging response suggests that even short‐duration (e.g., a few days, Figure S1) wet extremes can commonly reduce GPP across a variety of ecosystems.

**FIGURE 3:**
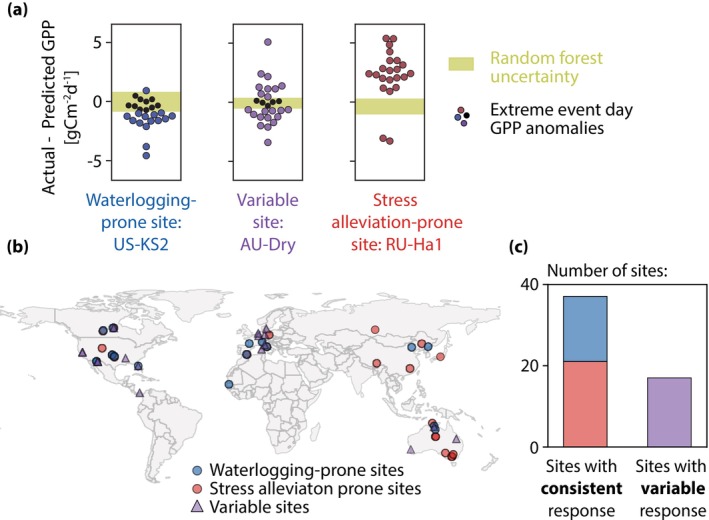
(a) Example for three sites that were categorized as waterlogging‐prone, variable, and stress alleviation‐prone. The dots represent individual extreme wet event day GPP anomalies (GPP_anom_) and the green band shows the random forest uncertainty. GPP_anom_ within the random forest uncertainty are shown in black and are excluded from further analysis. (b) Map of sites colored by category. Map lines delineate study areas and do not necessarily depict accepted national boundaries. (c) Bar chart showing the relative number of sites in each category.

While negative and positive GPP anomalies are about equally common across the dataset, at most individual sites the sign of the anomalies is less evenly split. Instead, extreme wet event day anomalies are often either all positive or all negative at a given site, regardless of storm severity. Example sites shown in Figure 3a demonstrate how GPP_anom_ at the waterlogging‐prone and stress alleviation‐prone sites are typically negative and positive, respectively. All sites are shown in Figure S6. Across all sites, 69% have consistent responses in the sign of the GPP response to extreme events, with stress alleviation‐prone sites (*n* = 21) being more common than waterlogging‐prone sites (*n* = 16) (Figure 3c). This categorization is not sensitive to the site record length, the random forest model performance, or the soil moisture threshold used to classify a day as “extreme” (Figure S7). Waterlogging‐prone sites are distributed around the globe and not only concentrated in regions known to be susceptible to excess moisture (such as energy‐limited ecosystems at high latitudes; Ohta et al. 2014; Yang et al. 2023) (Figure 3b). Furthermore, several sites that are located very close to each other—and therefore likely to experience the same or similar precipitation—nevertheless diverge in their response to extreme wet events, further highlighting the importance of site characteristics over storm conditions.

**FIGURE 4:**
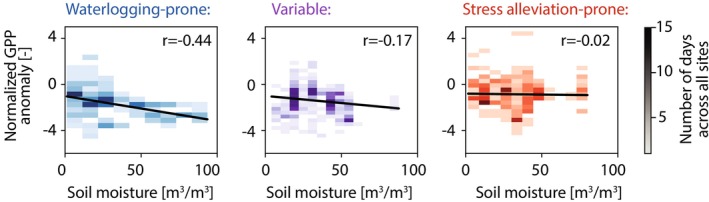
Two‐dimensional histograms showing the relationship between soil moisture (m^3^/m^3^) and the normalized GPP anomaly (GPP_anom,norm_). Darker colors indicate more days in each bin.

Our interpretation of the site categories is supported by the weakened—and sometimes reversed—relationship between GPP_anom,norm_ and extreme storm conditions at stress alleviation‐prone sites relative to waterlogging‐prone and variable sites. At waterlogging‐prone sites, higher soil moisture has a strongly negative correlation with GPP_anom,norm_ as expected (Figure 4). However, stress alleviation‐prone sites show a much weaker relationship between soil moisture and GPP_anom,norm_, consistent with our interpretation of excess soil moisture alleviating water stress and potentially even increasing GPP rather than inducing waterlogging. Stress alleviation‐prone sites show a weaker negative (or even a positive) GPP response when comparing GPP_anom,norm_ to other storm metrics as well, including the storm duration up to the measurement day, the antecedent soil moisture prior to the storm onset, and the maximum storm wind speed (Figure S8). This highlights that even extremely wet moisture conditions have the potential to either heighten or suppress GPP depending on site characteristics. Notably, the response of GPP_anom,norm_ at variable sites more closely resembles that of waterlogging‐prone sites than that of stress alleviation‐prone sites, with GPP suppression intensifying along with storm intensity. The different behavior of waterlogging‐prone, variable, and stress alleviation‐prone sites to increasingly extreme wet events raises a question about the factors that determine the category to which a site belongs.

**FIGURE 5:**
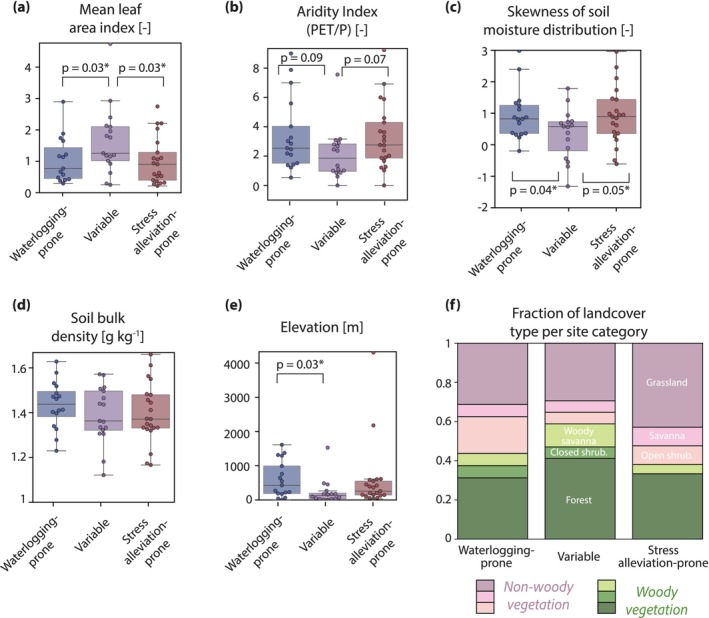
(a–e) Boxplots comparing site characteristics across waterlogging‐prone, variable, and stress alleviation‐prone sites. Significant differences in the mean across site distributions (using *t*‐test) are annotated and all other *p* values are provided in Table S2. Additional characteristics are shown in Figure S7. (f) The fraction of each category made up by woody (green) and non‐woody (pink) landcover types.

No single site characteristic fully explains whether a site is waterlogging‐prone, variable, or stress alleviation‐prone (Figure 5, see Figure S7 for more characteristics). None of the site characteristics significantly differ between waterlogging‐prone and stress alleviation‐prone sites. However, site mean leaf area index is significantly lower for waterlogging‐prone and stress alleviation‐prone sites than for variable sites (*p* = 0.03, *t*‐test) and the skewness of the soil moisture distribution is significantly higher for waterlogging‐prone and stress alleviation‐prone sites than for variable sites (*p* = 0.04, *t*‐test). Elevation at waterlogging‐prone sites is likewise statistically higher than at variable sites (*p*‐0.03, *t*‐test). Aside from these variables, however, no other site characteristic significantly differs between site categories (see Table S2 for all *p* values). The distribution of land cover types is also consistent between waterlogging‐prone and stress alleviation‐prone sites (*p* = 0.99, chi‐squared test) and between waterlogging‐ and stress alleviation‐prone sites compared to variable sites (*p* = 0.38 and 0.25, chi‐squared test). However, variable sites do have a higher incidence of woody vegetation (63% of sites) compared with waterlogging‐prone and stress alleviation‐prone sites (41% and 38%, respectively, Figure 5e). We also compare site categories to the species‐specific “tolerance to waterlogging” trait (see Section 2) and this, too, is unable to distinguish site categories (Figure S7) for the 13 genuses (across 21 out of 56 sites) where FLUXNET provides information on the dominant species and where those species are present in the TRY database (Kattge et al. 2011). Instead, all species are classified as intolerant to waterlogging regardless of the site category.

## 4 Discussion

We initially hypothesized that the storm characteristics—which reflect the degree of excess soil moisture and thus the potential for and degree of waterlogging—would be the primary determinant of GPP response to a given wet extreme. Instead, the most important variables for predicting the GPP anomaly were those capturing site characteristics. Indeed, the majority of sites have a consistent response to extreme wet events where GPP either decreases or increases relative to the expected baseline across all storms, regardless of intensity. When storms do become more intense, it is those sites that are waterlogging‐prone that exhibit increased negative GPP response, whereas the stress alleviation‐prone sites appear to be minimally affected—or even to benefit—from ever more extreme soil moisture.

The importance of site characteristics for the response of photosynthesis to wet extremes suggests that increases in the frequency and intensity of extreme storms under climate change will not impact all locations equally. Site characteristics could therefore be useful for both categorizing waterlogging risk and studying potential mitigation approaches. For example, growing investment in nature‐based climate solutions requires understanding limitations to vegetation growth and disturbance risks, particularly under future climate scenarios (Anderegg et al. 2020; Seddon 2022). Our work highlights that wet extremes—in addition to drought and wildfire—should be included in efforts to understand disturbance risks for nature‐based climate solutions. Wet extremes have the potential to meaningfully reduce global vegetation carbon uptake, as waterlogging‐prone locations represent nearly one‐third of the sites studied here (Figure 3) and are distributed across vegetation density, land cover type, and climate (Figure 4). In addition to reducing the land carbon sink, decreasing photosynthesis is also linked to reductions in transpiration (and therefore latent heat), resulting in additional impacts on local hydrology and land‐atmosphere interactions (Green et al. 2017).

To further understand how wet extremes affect photosynthesis, it is necessary to understand which site characteristics have a significant influence, in addition to the relative importance of site versus storm characteristics. Leaf area index, soil texture, and elevation all statistically differentiate waterlogging‐prone and stress alleviation‐prone sites from variable sites, but none of these significantly differentiates waterlogging‐prone and stress alleviation‐prone sites from each other (Figure 5). However, leaf area index, followed by soil texture, consistently dominates as the most important feature for explaining the GPP_anom,norm_ across all sites (Figure 2). Since leaf area index is related to vegetation type and density, and the soil texture and distribution metrics capture information about infiltration behavior, these characteristics likely integrate relevant information about vegetation response and the likelihood of inducing waterlogging from an extreme storm, respectively. Poorer‐draining soils have previously been associated with increased likelihood of waterlogging (Liu et al. 2023; Terazawa et al. 1992). Vegetation waterlogging response also differs between land cover types and species (Dreyer et al. 1991; Froend et al. 1987; Jentsch et al. 2009; Kreyling, Wenigmann, et al. 2008), in part due to genetic or morphological adaptations (e.g., adventitious rooting) that facilitate waterlogging tolerance (Black 1984; Dat and Parent 2012).

We find that climate and topography, two types of site characteristics that we would expect to constrain typical soil moisture states, and thus waterlogging likelihood, are less important for explaining GPP_anom,norm_ and for explaining the site categorization. However, topographic position has previously been found to explain how moisture variability manifests as vegetation waterlogging or drought response in the Amazon (Mattos et al. 2023). One explanation for the increased importance of vegetation density and soil characteristics over topography in this study may be that FLUXNET eddy covariance sites are typically placed in flat areas, and therefore may not capture the locations where topography matters most.

Although site characteristics are more important than storm conditions for explaining vegetation response, no single site characteristic differentiates waterlogging‐prone from stress alleviation‐prone sites. This inability to predict whether a site will be waterlogging‐prone or stress alleviation‐prone from individual site characteristics is in contrast to the high predictive power of these same site characteristics (even without storm information) to predict the magnitude of the GPP anomaly. This may be because the magnitude of the GPP anomaly depends on the interactions of different site characteristics in a nonlinear way. To achieve the goal of predicting and managing the impacts from extreme storms, we therefore need to better understand the mechanisms and interactions by which site characteristics affect vegetation response. However, there are few in situ studies on the effects of short‐lived extreme wet events. The effects of saturated soil are commonly studied in potted plants in greenhouses (e.g., Dreyer et al. 1991; Malik et al. 2002; Terazawa et al. 1992), but such experiments are unable to capture soil or topographic effects or interactions among individuals. When experiments are conducted in natural settings, they commonly target conditions with prolonged waterlogging, such as wetlands or marshes where salinity and vegetation tolerance matter in addition to soil saturation (e.g., Conner 1994; Froend et al. 1987; Kozlowski 1997), or synthesize the effects of large and sudden inundation such as that induced by dam construction (Black 1984; Whitlow and Harris 1979). This hampers our ability to relate our findings here to mechanistic explanations about plant physiology. Likewise, estimates of species‐specific tolerance to waterlogging are typically assessed under long‐lasting flooding or riparian conditions (Whitlow and Harris 1979). This may explain why the “tolerance to waterlogging” trait in TRY was identical for all of the species with trait information available at our sites despite our finding that leaf area index is the most explanatory site characteristic for waterlogging prevalence. The work presented here therefore illustrates the need for additional experiments that identify the physiological drivers for photosynthesis reductions under sudden and severe waterlogging. A necessary but natural component of understanding these drivers may be the development of alternative measurable traits that can serve to quantify relative sensitivity to waterlogging across species in a more granular way than the current “tolerance to waterlogging” trait in TRY.

The anomaly calculation approach presented here provides a framework for evaluating future modeling work on the impact of soil saturation on photosynthesis. Photosynthesis suppression as a function of soil dryness has been represented in land surface models via a multiplicative “beta” function for decades (Trugman et al. 2018). By contrast, photosynthesis suppression in response to waterlogging has only been recently included in some agricultural models (Liu et al. 2023) but is not currently represented in global land surface models (Trugman et al. 2018). Furthermore, the importance of site characteristics implies that a single multiplicative response function applied regardless of location (as most models currently do to simulate drought response; Trugman et al. 2018) is unlikely to fully capture photosynthesis response to wet extremes. Our approach could be used to test model predictions in the face of complex responses and compensating errors associated with the isolation of vegetation responses to a variety of extreme conditions. The results presented here will aid future experimental and theoretical work, which is needed for developing frameworks and identifying the mechanisms responsible for photosynthesis response under specific storm and site characteristics. Our work suggests that understanding the role of interacting site characteristics, in particular, is needed to predict why some locations benefit from extreme storms while others suffer.

## Author Contributions

**Erica L. McCormick:** conceptualization, data curation, formal analysis, investigation, methodology, software, visualization, writing – original draft, writing – review and editing. **Caroline A. Famiglietti:** conceptualization, methodology. **Dapeng Feng:** investigation, methodology. **Anna M. Michalak:** conceptualization, investigation, methodology, supervision, visualization, writing – review and editing. **Alexandra G. Konings:** conceptualization, funding acquisition, investigation, methodology, project administration, resources, supervision, visualization, writing – original draft, writing – review and editing.

## Conflicts of Interest

The authors declare no conflicts of interest.

## Supporting information


Appendix S1.

## Data Availability

The data and code that support the findings of this study are openly available in Zenodo at https://doi.org/10.5281/zenodo.15360984. The FLUXNET2015 dataset was obtained from https://fluxnet.org/data/fluxnet2015‐dataset/.
