# Supplementary material for: Susceptibility to Photosynthesis Suppression From Extreme Storms Is Highly Site‐Dependent
Source: Glob Chang Biol. 2025 May 22;31(5):e70257. doi: 10.1111/gcb.70257 (PMC12096146; doi:10.1111/gcb.70257)
Supplement: Supplementary file 1 — Appendix S1. [file GCB-31-e70257-s001.pdf]

Supplemental figures for
"Susceptibility to photosynthesis suppression from extreme storms is highly site-dependent"

Erica L. McCormick[1], Caroline A. Famiglietti[1,2], Dapeng Feng[1,3], Anna M. Michalak[1,4], and Alexandra G. Konings[1]

[1] Department of Earth System Science, Stanford University
[2] Hydrosat, Inc.
[3] Stanford Institute for Human-Centered Artificial Intelligence (HAI), Stanford University
[4] Department of Global Ecology, Carnegie Institution for Science

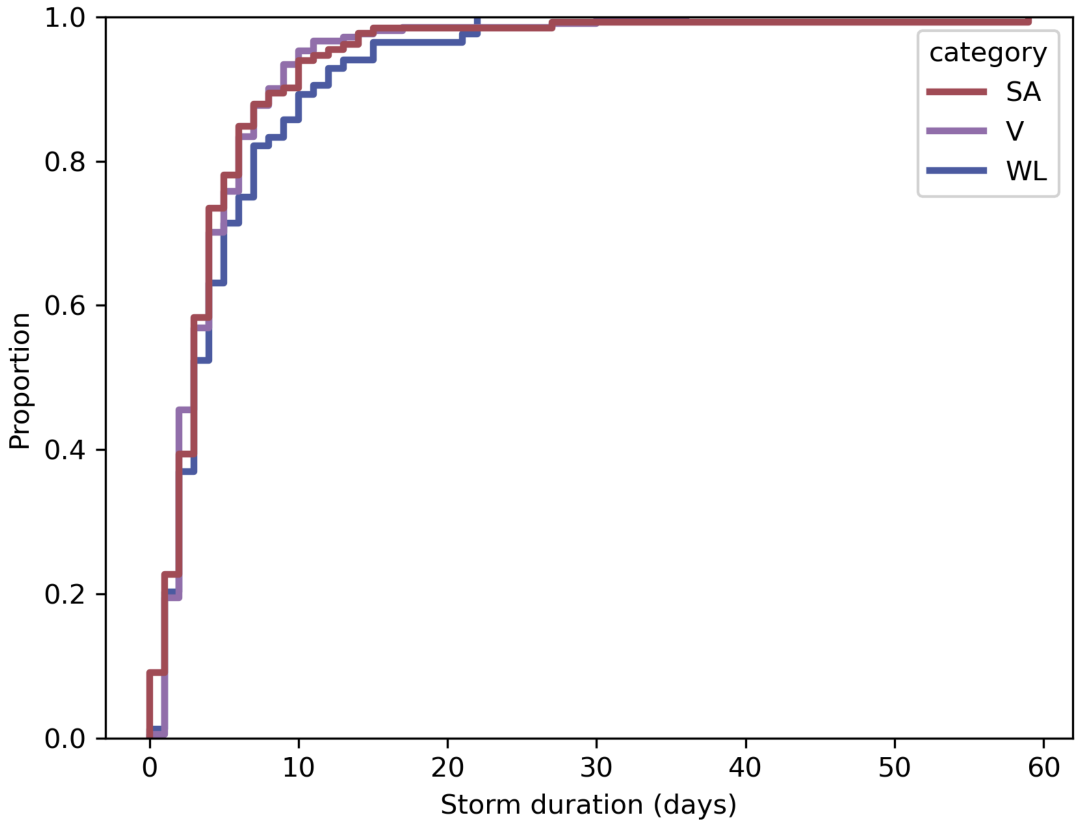

Figure S1. CDF of storm duration in days grouped by site categories of waterlogging-prone (WL), water stress alleviation-prone (SA), and variable (V). 87% of storms in our dataset last less than or equal to 1 week and 56% are less than or equal to 3 days. Storm durations are similar across site categories.

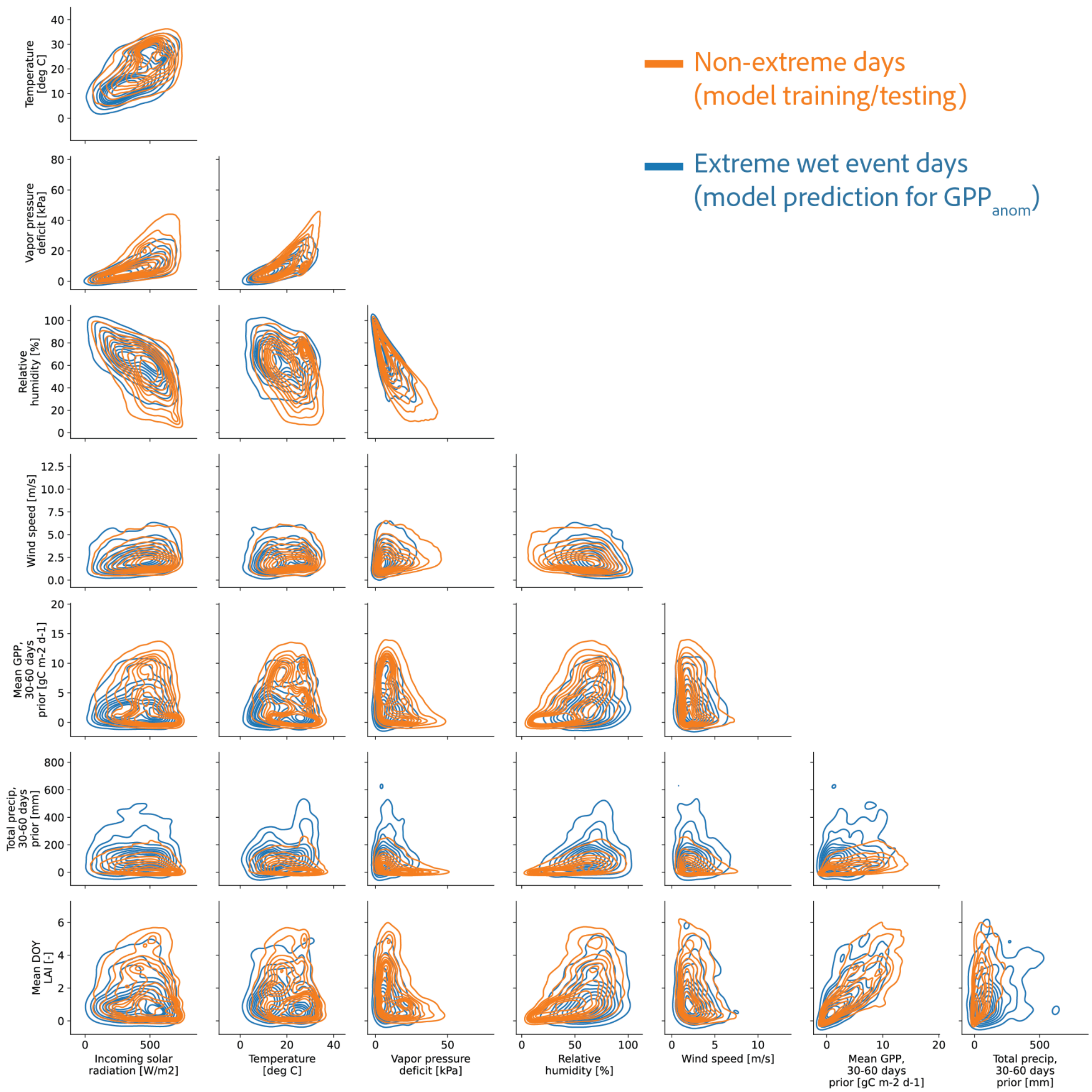

Figure S2. Cross correlations between feature inputs for the random forest model to predict GPP. Distributions are shown for the extreme wet event day (blue) and for the non-extreme days used for model training and testing (orange).

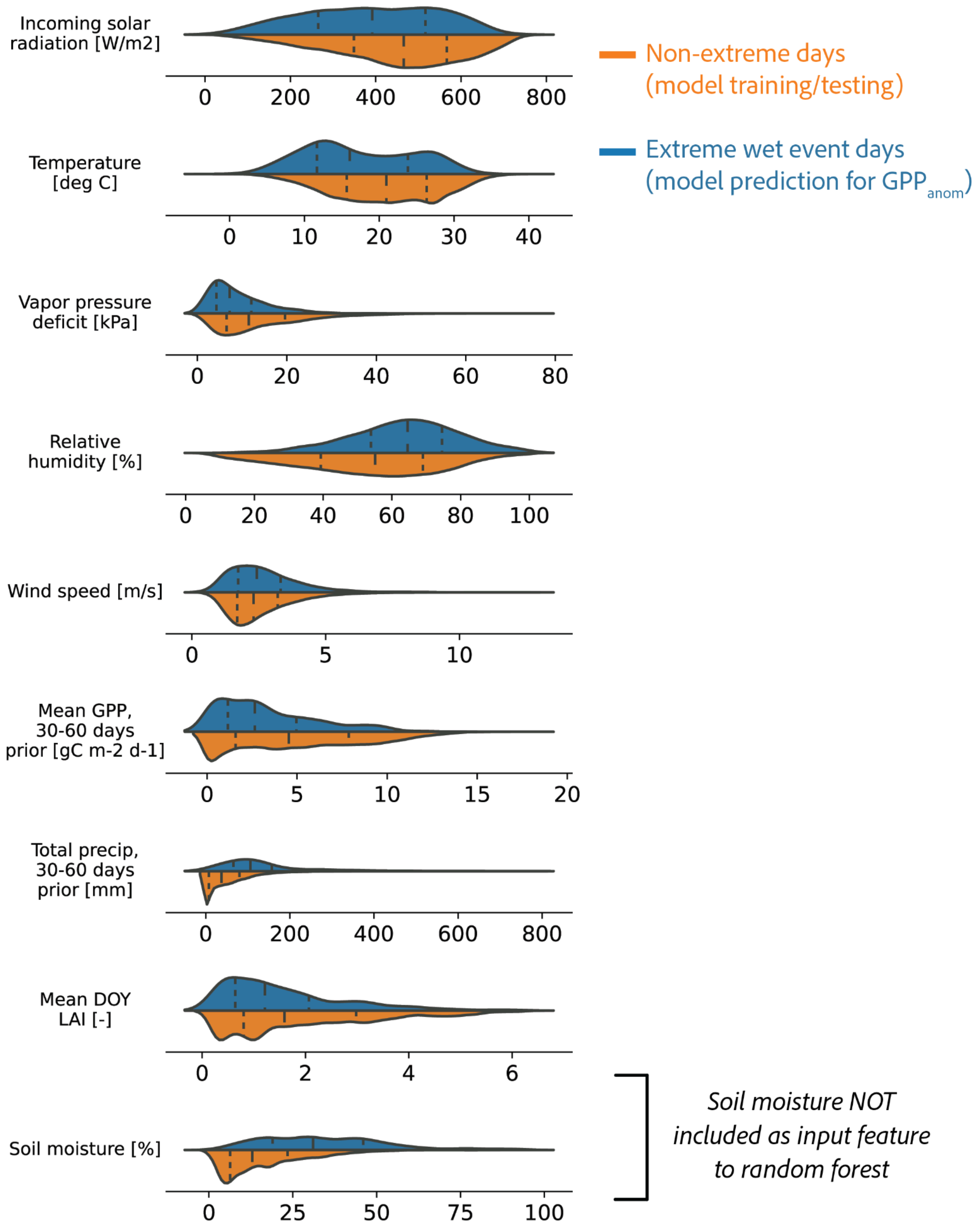

Figure S3. Violinplot distributions for the feature inputs to the random forest model to predict GPP plus the distribution of soil moisture. Dashed lines represent the mean and interquartile range, respectively. Distributions are shown for the extreme wet event days (blue) and for the non-extreme days used for model training and testing (orange).

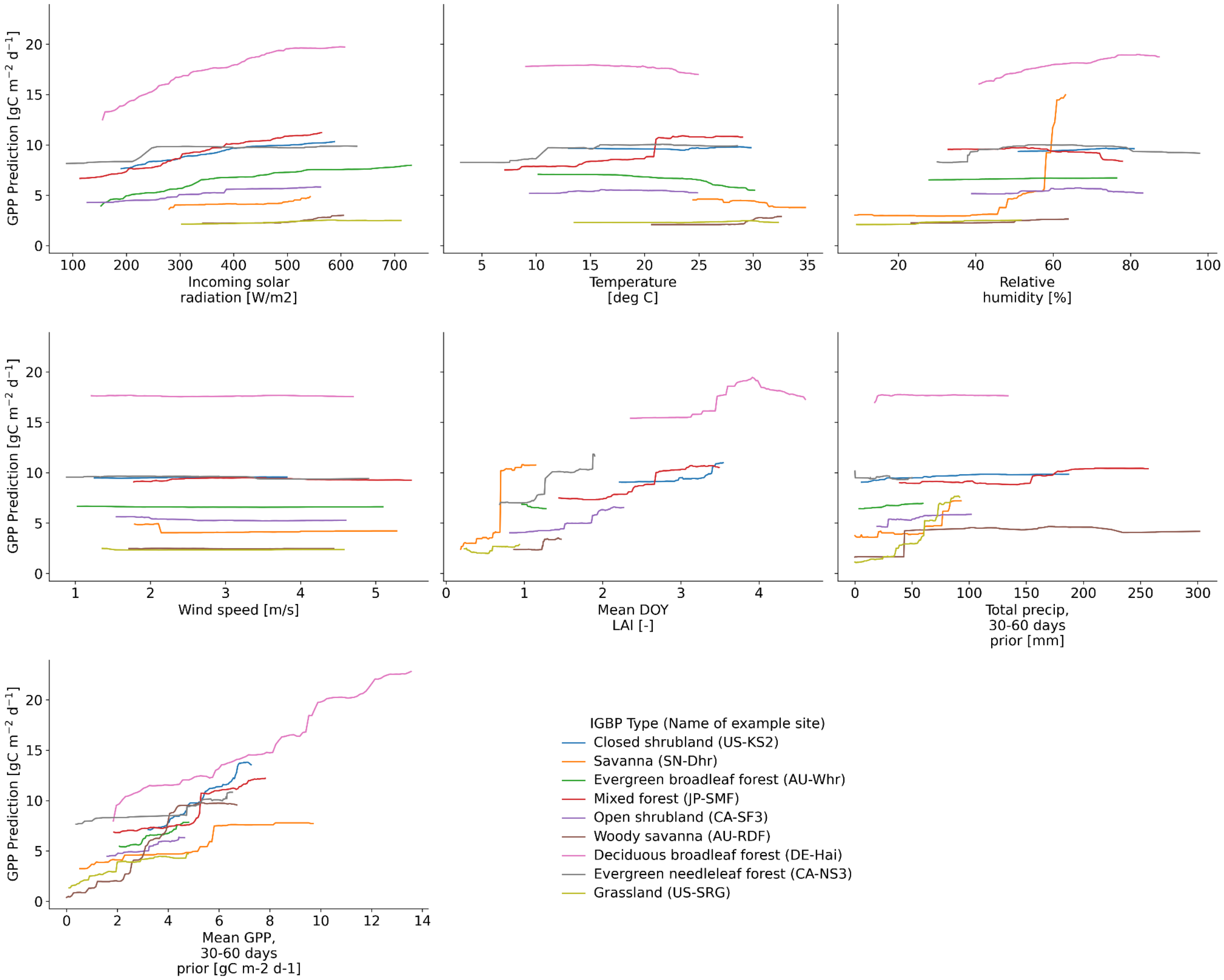

Figure S4. Partial dependence plots for input features to random forest model to predict GPP. Because there is an individual model for each site, one example site is shown for each landcover type.

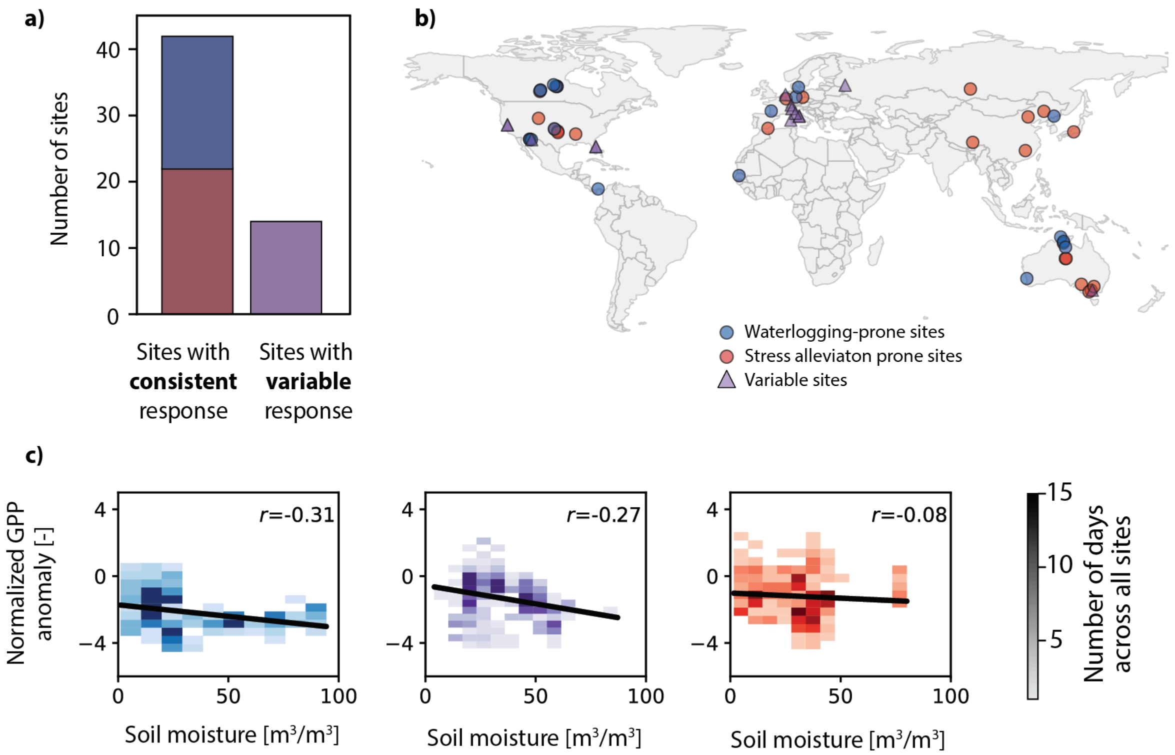

Figure S5. Key results using GPP from the nighttime partitioning method. (a) Bar chart showing the relative number of sites categorized as waterlogging-prone (blue), stress alleviation-prone (red) or variable (purple). (b) Map of sites colored by category. (c) 2D histograms of soil moisture on the day of the extreme event vs the normalized GPP anomaly ($GPP_{anom,norm}$). Darker colors indicate more days in each bin. $GPP_{anom,norm}$ is increasingly negative at waterlogging-prone and variable sites with increasing soil moisture, in contrast to stress alleviation-prone sites, which have a weaker relationship between $GPP_{anom,norm}$ and soil moisture.

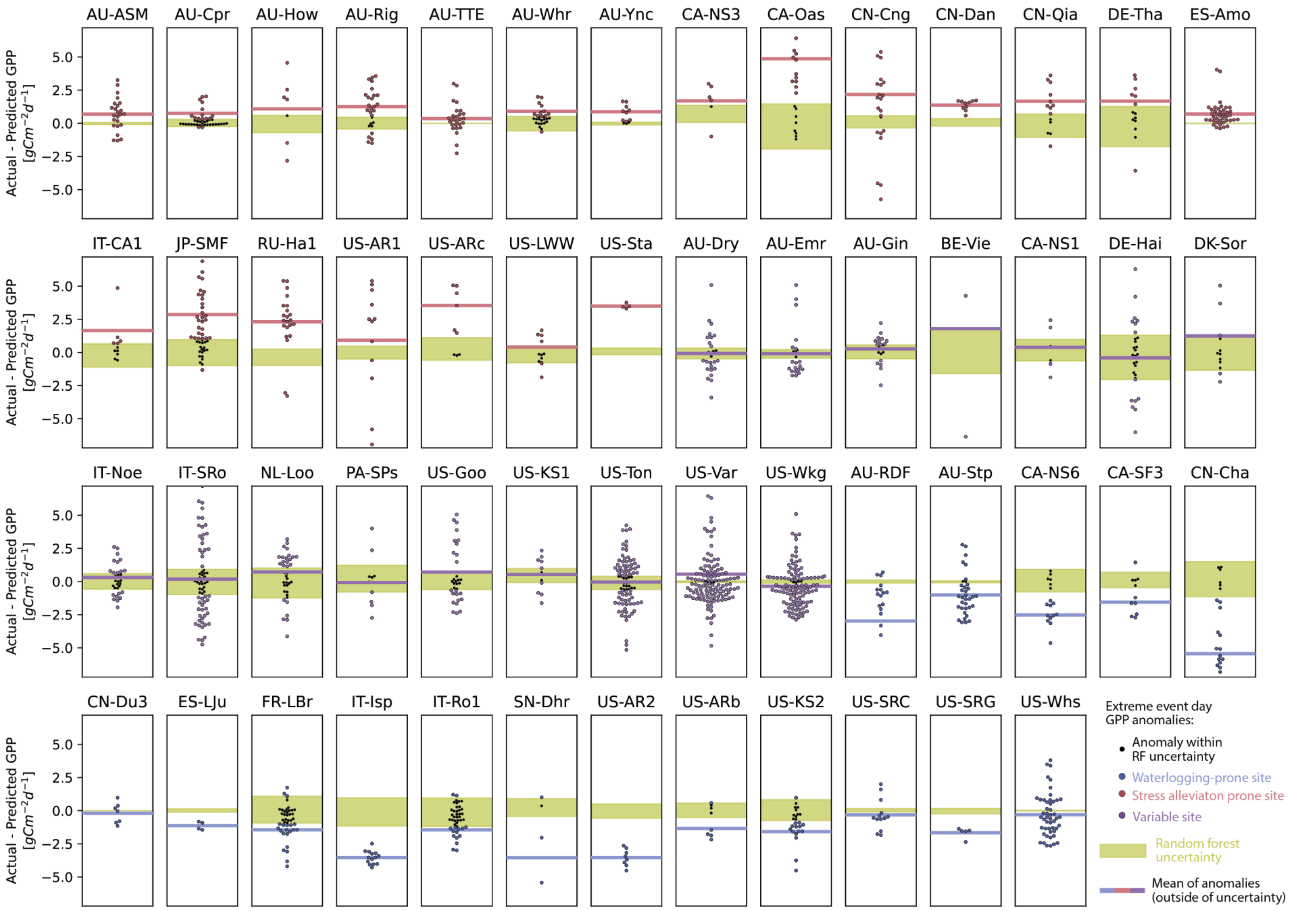

Figure S6. All extreme wet event day GPP anomalies (GPP_{anom}) at all sites. Green shading shows the random forest uncertainty (calculated as the 25th to 75th percentile of the GPP anomalies on the withheld, non-extreme test days) and the colored bar is the mean of the extreme event day anomalies outside of this uncertainty. Black dots are extreme event day anomalies within the random forest uncertainty, which are excluded from further analysis. Blue, red, and purple coloring indicates whether the site is classified as waterlogging-prone, stress alleviation-prone, or variable, respectively, based on whether the mean of GPP_{anom} is less than, greater than, or within the random forest uncertainty (see Methods). US-Wkg was manually reclassified from waterlogging-prone to variable and US-Var was reclassified manually from stress alleviation-prone to variable.

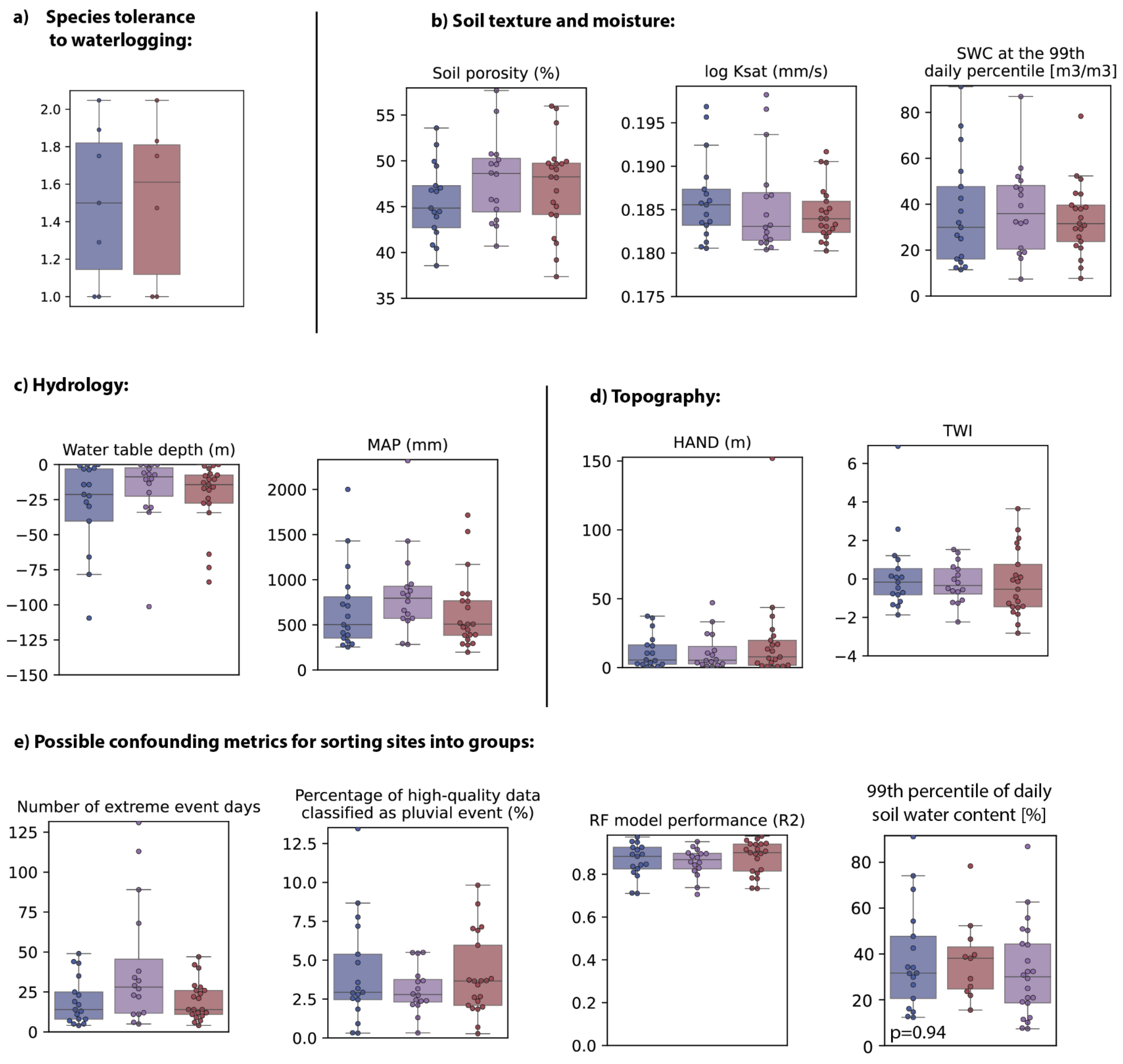

Figure S7. Site characteristics at waterlogging-prone, variable, and stress alleviation-prone sites. See Figure 4 in the main text for additional variables and Table S2 for the p-values between all site categories. (a) Mean tolerance to waterlogging trait from the TRY database (Kattge et al., 2011) for the 13 genuses (across 21 out of 56 sites) which are listed as a dominant species at FLUXNET site and which have information in TRY. This trait is measured on a scale from 1 to 5 (Whitlow & Harris, 1979) where 1 is “very intolerant (does not tolerate water-saturated soils for more than a few days during the growing season)”, 2 is “intolerant (tolerates one to two weeks of WL during the growing season” and extends to 5, very tolerant, where vegetation “survives deep, prolonged WL for more than one year.” (b) Soil texture for each site. (c) Hydrologic indicators of mean annual precipitation (MAP) and the depth to water table

(Miguez-Macho & Fan, 2021). Note that the depth to water table values represent large pixel areas which are modeled based on measurements, but do not necessarily represent actual values at the site scale. (d) Topographic metrics of height above nearest drainage (HAND) and topographic wetness index (TWI). (e) Site characteristics tested to check for association with waterlogging-prone and stress alleviation-prone categories, including the random forest model performance, the number of extreme event days, the number of high-quality data at the site, and the soil water content at the 99th percentile value at the site.

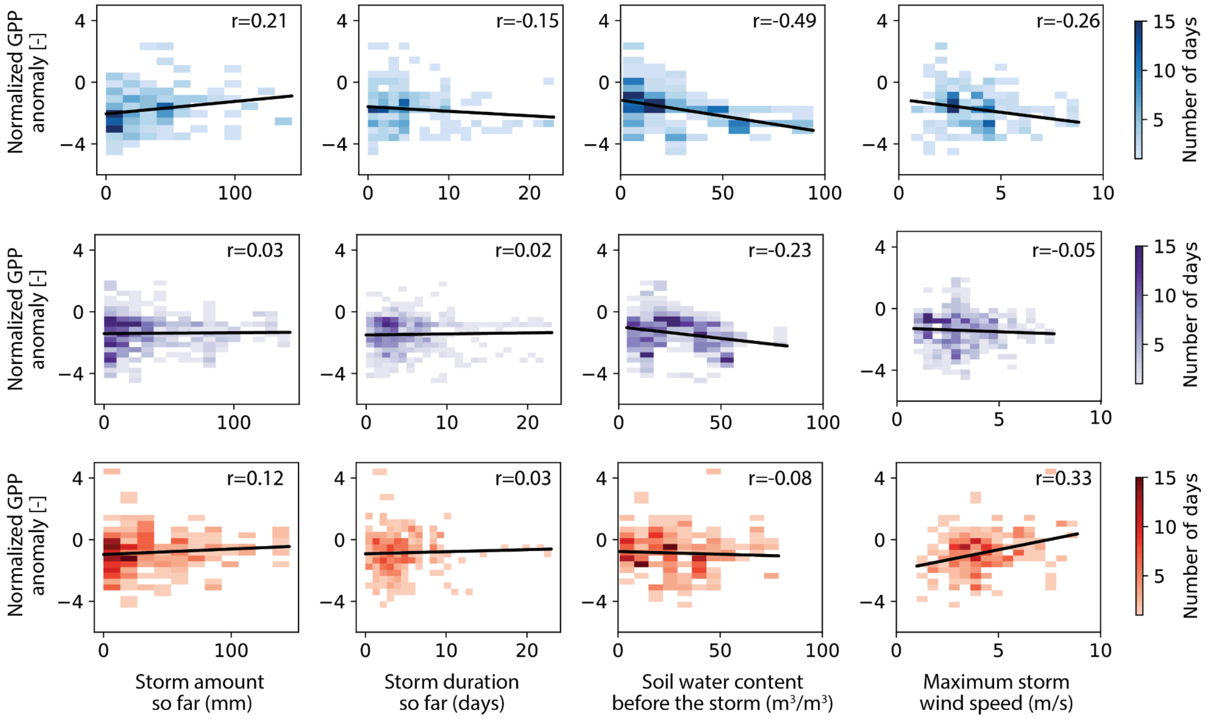

Figure S8. 2D histograms of the normalized GPP anomaly ($GPP_{anom,norm}$) across storm metrics of the cumulative precipitation during the storm up to the day of the measurement, the storm duration up to the day of the measurement, the soil water content before the storm, and the maximum storm wind speed.

Table S1. Site ID, location, random forest model performance, site category, number of extreme wet event days outside of the random forest uncertainty, and DOI. Sites are categorized as waterlogging-prone (WL), stress alleviation-prone (SA), or variable (V).

Site Name	Latitude	Longitude	Site Category	Model Performance (R^2)	Number of event days outside of model uncertainty	DOI
AU-ASM	-22.283	133.249	SA	0.97	24	10.18140/FLX/1440194
AU-Cpr	-34.0021	140.5891	SA	0.91	42	10.18140/FLX/1440195
AU-How	-12.4943	131.1523	SA	0.90	7	10.18140/FLX/1440125
AU-Rig	-36.6499	145.5759	SA	0.94	28	10.18140/FLX/1440202
AU-TTE	-22.287	133.64	SA	0.92	26	10.18140/FLX/1440205
AU-Whr	-36.6732	145.0294	SA	0.78	29	10.18140/FLX/1440206
AU-Ync	-34.9893	146.2907	SA	0.98	12	10.18140/FLX/1440208
CA-NS3	55.91167	-98.38222	SA	0.73	6	10.18140/FLX/1440038
CA-Oas	53.62889	-106.19779	SA	0.78	21	10.18140/FLX/1440043
CN-Cng	44.5934	123.5092	SA	0.90	26	10.18140/FLX/1440209
CN-Dan	30.4978	91.0664	SA	0.92	10	10.18140/FLX/1440138
CN-Qia	26.7414	115.0581	SA	0.80	14	10.18140/FLX/1440141
DE-Tha	50.96256	13.56515	SA	0.74	14	10.18140/FLX/1440152
ES-Amo	36.83361	-2.25232	SA	0.94	40	10.18140/FLX/1440156
IT-CA1	42.38041	12.02656	SA	0.90	11	10.18140/FLX/1440230
JP-SMF	35.2617	137.0788	SA	0.87	47	10.18140/FLX/1440239
RU-Ha1	54.72517	90.00215	SA	0.82	22	10.18140/FLX/1440184
US-AR1	36.4267	-99.42	SA	0.81	14	10.18140/FLX/1440103
US-ARc	35.54649	-98.04	SA	0.96	9	10.18140/FLX/1440065
US-LWW	34.9604	-97.9789	SA	0.94	12	10.18140/FLX/1440077
US-Sta	41.3966	-106.8024	SA	0.94	4	10.18140/FLX/1440115
AU-Dry	-15.2588	132.3706	V	0.90	27	10.18140/FLX/1440197
AU-Emr	-23.8587	148.4746	V	0.86	23	10.18140/FLX/1440198
AU-Gin	-31.3764	115.7138	V	0.85	22	10.18140/FLX/1440199
BE-Vie	50.30493	5.99812	V	0.74	5	10.18140/FLX/1440130
CA-NS1	55.87917	-98.48389	V	0.71	6	10.18140/FLX/1440036
DE-Hai	51.07921	10.45217	V	0.83	29	10.18140/FLX/1440148
DK-Sor	55.48587	11.64464	V	0.92	12	10.18140/FLX/1440155
IT-Noe	40.60618	8.15169	V	0.84	32	10.18140/FLX/1440171
IT-SRo	43.72786	10.28444	V	0.88	68	10.18140/FLX/1440176
NL-Loo	52.16658	5.74356	V	0.80	38	10.18140/FLX/1440178
PA-SPs	9.31378	-79.63143	V	0.93	11	10.18140/FLX/1440179
US-Goo	34.2547	-89.8735	V	0.88	34	10.18140/FLX/1440070
US-KS1	28.4583	-80.6709	V	0.82	11	10.18140/FLX/1440074
US-Ton	38.4316	-120.96598	V	0.90	89	10.18140/FLX/1440092
US-Var	38.4133	-120.9507	V	0.95	131	10.18140/FLX/1440094
US-Wkg	31.7365	-109.9419	V	0.90	113	10.18140/FLX/1440096
AU-RDF	-14.5636	132.4776	WL	0.97	19	10.18140/FLX/1440201
AU-Stp	-17.1507	133.3502	WL	0.95	35	10.18140/FLX/1440204
CA-NS6	55.91667	-98.96444	WL	0.81	17	10.18140/FLX/1440041
CA-SF3	54.09156	-106.00526	WL	0.71	11	10.18140/FLX/1440048
CN-Cha	42.4025	128.0958	WL	0.91	23	10.18140/FLX/1440137
CN-Du3	42.0551	116.2809	WL	0.84	7	10.18140/FLX/1440210
ES-LJu	36.92659	-2.75212	WL	0.93	4	10.18140/FLX/1440157
FR-LBr	44.71711	-0.7693	WL	0.85	44	10.18140/FLX/1440163
IT-Isp	45.81264	8.63358	WL	0.93	14	10.18140/FLX/1440234
IT-Ro1	42.40812	11.93001	WL	0.88	43	10.18140/FLX/1440174
SN-Dhr	15.40278	-15.43222	WL	0.89	5	10.18140/FLX/1440246
US-AR2	36.6358	-99.5975	WL	0.79	8	10.18140/FLX/1440104
US-ARb	35.5497	-98.0402	WL	0.95	8	10.18140/FLX/1440064
US-KS2	28.6086	-80.6715	WL	0.83	25	10.18140/FLX/1440075
US-SRC	31.9083	-110.8395	WL	0.71	13	10.18140/FLX/1440098
US-SRG	31.789379	-110.82768	WL	0.89	5	10.18140/FLX/1440114
US-Whs	31.7438	-110.0522	WL	0.84	49	10.18140/FLX/1440097

Table S2. P-values comparing the mean site characteristics across site categories (t-test). Boxplots are shown in Figure 5 and Figure S7. Significant p-values (<0.05) are highlighted in green.

Site Characteristic	Waterlogging-prone vs Stress alleviation-prone	Waterlogging-prone vs Variable	Stress alleviation-prone vs Variable
Soil bulk density (g/kg)	0.24	0.13	0.70
Soil porosity (%)	0.24	0.13	0.70
Soil surface hydraulic conductivity (mm/sec)	0.26	0.76	0.54
Skewness of soil moisture distribution (-)	0.92	0.04	0.05
Soil moisture at 99th percentile (%)	0.73	0.84	0.53
Height above nearest drainage (HAND) (-)	0.37	1.00	0.38
Topographic wetness index (-)	0.49	0.46	0.98
Elevation (m)	0.91	0.03	0.16
Mean leaf area index	0.94	0.03	0.03
Water table depth (m)	0.27	0.19	0.56
Mean annual temperature (deg C)	0.30	0.97	0.31
Mean annual precipitation (mm)	0.75	0.31	0.16
Aridity (-)	0.90	0.09	0.08
Random forest model performance (R2) (-)	0.57	0.75	0.35
Number of extreme event days	0.91	0.05	0.05
Number of extreme event days / number of test days	0.85	0.24	0.19

References

Berbigier, P., Bonnefond, J.-M., & Mellmann, P. (2001). CO2 and water vapour fluxes for 2 years above Euroflux forest site. *Agricultural and Forest Meteorology*, *108*(3), 183–197. https://doi.org/10.1016/S0168-1923(01)00240-4

Beringer, J., Hutley, L. B., Hacker, J. M., Neininger, B., & Paw U, K. T. (2011). Patterns and processes of carbon, water and energy cycles across northern Australian landscapes: From point to region. *Agricultural and Forest Meteorology*, *151*(11), 1409–1416. https://doi.org/10.1016/j.agrformet.2011.05.003

Beringer, J., Hutley, L. B., McHugh, I., Arndt, S. K., Campbell, D., Cleugh, H. A., Cleverly, J., Resco De Dios, V., Eamus, D., Evans, B., Ewenz, C., Grace, P., Griebel, A., Haverd, V., Hinko-Najera, N., Huete, A., Isaac, P., Kanniah, K., Leuning, R., … Wardlaw, T. (2016). An introduction to the Australian and New Zealand flux tower network –OzFlux. *Biogeosciences*, *13*(21), 5895–5916. https://doi.org/10.5194/bg-13-5895-2016

Beringer, J., Hutley, L. B., Tapper, N. J., & Cernusak, L. A. (2007). Savanna fires and their impact on net ecosystem productivity in North Australia. *Global Change Biology*, *13*(5), 990–1004. https://doi.org/10.1111/j.1365-2486.2007.01334.x

Bristow, M., Hutley, L. B., Beringer, J., Livesley, S. J., Edwards, A. C., & Arndt, S. K. (2016). Quantifying the relative importance of greenhouse gas emissions from current and future savanna land use change across northern Australia. *Biogeosciences*, *13*(22), 6285–6303. https://doi.org/10.5194/bg-13-6285-2016

Cernusak, L. A., Hutley, L. B., Beringer, J., Holtum, J. A. M., & Turner, B. L. (2011). Photosynthetic physiology of eucalypts along a sub-continental rainfall gradient in northern Australia. *Agricultural and Forest Meteorology*, *151*(11), 1462–1470. https://doi.org/10.1016/j.agrformet.2011.01.006

Chiesi, M., Maselli, F., Bindi, M., Fibbi, L., Cherubini, P., Arlotta, E., Tirone, G., Matteucci, G., & Seufert, G. (2005). Modelling carbon budget of Mediterranean forests using ground and remote sensing measurements. *Agricultural and Forest Meteorology*, *135*(1–4), 22–34. https://doi.org/10.1016/j.agrformet.2005.09.011

Chu, H., Baldocchi, D. D., Poindexter, C., Abraha, M., Desai, A. R., Bohrer, G., Arain, M. A., Griffis, T., Blanken, P. D., O'Halloran, T. L., Thomas, R. Q., Zhang, Q., Burns, S. P., Frank, J. M., Christian, D., Brown, S., Black, T. A., Gough, C. M., Law, B. E., … Martin, T. A. (2018). Temporal Dynamics of Aerodynamic Canopy Height Derived From Eddy Covariance Momentum Flux Data Across North American Flux Networks. *Geophysical Research Letters*, *45*(17), 9275–9287. https://doi.org/10.1029/2018GL079306

Cleverly, J., Boulain, N., Villalobos-Vega, R., Grant, N., Faux, R., Wood, C., Cook, P. G., Yu, Q., Leigh, A., & Eamus, D. (2013). Dynamics of component carbon fluxes in a semi-arid *Acacia* woodland, central Australia. *Journal of Geophysical Research: Biogeosciences*, *118*(3), 1168–1185. https://doi.org/10.1002/jgrg.20101

Ferréa, C., Zenone, T., Comolli, R., & Seufert, G. (2012). Estimating heterotrophic and autotrophic soil respiration in a semi-natural forest of Lombardy, Italy. *Pedobiologia*, *55*(6), 285–294. https://doi.org/10.1016/j.pedobi.2012.05.001

Fischer, M. L., Torn, M. S., Billesbach, D. P., Doyle, G., Northup, B., & Biraud, S. C. (2012). Carbon, water, and heat flux responses to experimental burning and drought in a tallgrass prairie. *Agricultural and Forest Meteorology*, *166–167*, 169–174. https://doi.org/10.1016/j.agrformet.2012.07.011

Guan, D.-X., Wu, J.-B., Zhao, X.-S., Han, S.-J., Yu, G.-R., Sun, X.-M., & Jin, C.-J. (2006). CO2 fluxes over an old, temperate mixed forest in northeastern China. *Agricultural and Forest Meteorology*, *137*(3–4), 138–149. https://doi.org/10.1016/j.agrformet.2006.02.003

Kattge, J., Diaz, S., Lavorel, S., Prentice, C., Leadley, P., Boenisch, G., Garnier, E., Westoby, M., Reich, P. B., Wright, I. J., Cornelissen, J. H. C., Violle, C., Harrison, S. P., van Bodegom, P. M., Reichstein, M., Enquist, B. J., Soudzilovskaia, N. A., Ackerly, D. D., Anand, M., … Wirth, C. (2011). TRY - a global database of plant traits. Global Change Biology, 17(9), 2905–2935. https://doi.org/10.1111/j.1365-2486.2011.02451.x

López-Ballesteros, A., Serrano-Ortiz, P., Kowalski, A. S., Sánchez-Cañete, E. P., Scott, R. L., & Domingo, F. (2017). Subterranean ventilation of allochthonous CO 2 governs net CO 2 exchange in a semiarid Mediterranean grassland. *Agricultural and Forest Meteorology*, *234–235*, 115–126. https://doi.org/10.1016/j.agrformet.2016.12.021

Marchesini, L. B., Papale, D., Reichstein, M., Vuichard, N., Tchebakova, N., & Valentini, R. (2007). *Carbon balance assessment of a natural steppe of southern Siberia by multiple constraint approach*.

Matsumoto, K., Ohta, T., Nakai, T., Kuwada, T., Daikoku, K., Iida, S., Yabuki, H., Kononov, A. V., Van Der Molen, M. K., Kodama, Y., Maximov, T. C., Dolman, A. J., & Hattori, S. (2008). Energy consumption and evapotranspiration at several boreal and temperate forests in the Far East. *Agricultural and Forest Meteorology*, *148*(12), 1978–1989. https://doi.org/10.1016/j.agrformet.2008.09.008

McHugh, I. D., Beringer, J., Cunningham, S. C., Baker, P. J., Cavagnaro, T. R., Mac Nally, R., & Thompson, R. M. (2017). Interactions between nocturnal turbulent flux, storage and advection at an "ideal" eucalypt woodland site. *Biogeosciences*, *14*(12), 3027–3050. https://doi.org/10.5194/bg-14-3027-2017

Meyer, W. S., Kondrlovà, E., & Koerber, G. R. (2015). Evaporation of perennial semi-arid woodland in southeastern Australia is adapted for irregular but common dry periods. *Hydrological Processes*, *29*(17), 3714–3726. https://doi.org/10.1002/hyp.10467

Miguez-Macho, G., & Fan, Y. (2021). Spatiotemporal origin of soil water taken up by vegetation. Nature, 598(7882), 624–628. https://doi.org/10.1038/s41586-021-03958-6

Pilegaard, K., Ibrom, A., Courtney, M. S., Hummelshøj, P., & Jensen, N. O. (2011). Increasing net CO2 uptake by a Danish beech forest during the period from 1996 to 2009. *Agricultural and Forest Meteorology*, *151*(7), 934–946. https://doi.org/10.1016/j.agrformet.2011.02.013

Powell, T. L., Bracho, R., Li, J., Dore, S., Hinkle, C. R., & Drake, B. G. (2006). Environmental controls over net ecosystem carbon exchange of scrub oak in central Florida. *Agricultural and Forest Meteorology*, *141*(1), 19–34. https://doi.org/10.1016/j.agrformet.2006.09.002

Reed, D. E., Ewers, B. E., Pendall, E., Naithani, K. J., Kwon, H., & Kelly, R. D. (2018). Biophysical Factors and Canopy Coupling Control Ecosystem Water and Carbon Fluxes of Semiarid Sagebrush Ecosystems. *Rangeland Ecology & Management*, *71*(3), 309–317. https://doi.org/10.1016/j.rama.2018.01.003

Rey, A., Pegoraro, E., Tedeschi, V., De Parri, I., Jarvis, P. G., & Valentini, R. (2002). Annual variation in soil respiration and its components in a coppice oak forest in Central Italy. *Global Change Biology*, *8*(9), 851–866. https://doi.org/10.1046/j.1365-2486.2002.00521.x

Runkle, B. R. K., Rigby, J. R., Reba, M. L., Anapalli, S. S., Bhattacharjee, J., Krauss, K. W., Liang, L., Locke, M. A., Novick, K. A., Sui, R., Suvočarev, K., & White, P. M. (2017). Delta-Flux: An Eddy Covariance Network for a Climate-Smart Lower Mississippi Basin. *Agricultural & Environmental Letters*, *2*(1), ael2017.01.0003. https://doi.org/10.2134/ael2017.01.0003

Sabbatini, S., Arriga, N., Bertolini, T., Castaldi, S., Chiti, T., Consalvo, C., Njakou Djomo, S., Gioli, B., Matteucci, G., & Papale, D. (2016). Greenhouse gas balance of cropland conversion to bioenergy poplar short-rotation coppice. *Biogeosciences*, *13*(1), 95–113. https://doi.org/10.5194/bg-13-95-2016

Serrano-Ortiz, P., Domingo, F., Cazorla, A., Were, A., Cuezva, S., Villagarcía, L., Alados-Arboledas, L., & Kowalski, A. S. (2009). Interannual CO_2 exchange of a sparse Mediterranean shrubland on a carbonaceous substrate. *Journal of Geophysical Research: Biogeosciences*, *114*(G4), 2009JG000983. https://doi.org/10.1029/2009JG000983

Shi, P., Sun, X., Xu, L., Zhang, X., He, Y., Zhang, D., & Yu, G. (2006). Net ecosystem CO2 exchange and controlling factors in a steppe—Kobresia

meadow on the Tibetan Plateau. *Science in China Series D: Earth Sciences*, *49*(S2), 207–218. https://doi.org/10.1007/s11430-006-8207-4

Sluis, W. J. (2002). Patterns of Species Richness and Composition in Re-Created Grassland. *Restoration Ecology*, *10*(4), 677–684. https://doi.org/10.1046/j.1526-100X.2002.01048.x

Spano, D., Snyder, R. L., Sirca, C., & Duce, P. (2009). ECOWAT—A model for ecosystem evapotranspiration estimation. *Agricultural and Forest Meteorology*, *149*(10), 1584–1596. https://doi.org/10.1016/j.agrformet.2009.04.011

Steele, S. J., Gower, S. T., Vogel, J. G., & Norman, J. M. (1997). Root mass, net primary production and turnover in aspen, jack pine and black spruce forests in Saskatchewan and Manitoba, Canada. *Tree Physiology*, *17*(8–9), 577–587. https://doi.org/10.1093/treephys/17.8-9.577

Tagesson, T., Fensholt, R., Guiro, I., Rasmussen, M. O., Huber, S., Mbow, C., Garcia, M., Horion, S., Sandholt, I., Holm-Rasmussen, B., Göttsche, F. M., Ridler, M., Olén, N., Lundegard Olsen, J., Ehammer, A., Madsen, M., Olesen, F. S., & Ardö, J. (2015). Ecosystem properties of semiarid savanna grassland in West Africa and its relationship with environmental variability. *Global Change Biology*, *21*(1), 250–264. https://doi.org/10.1111/gcb.12734

Twine, T. E., Kustas, W. P., Norman, J. M., Cook, D. R., Houser, P. R., Meyers, T. P., Prueger, J. H., Starks, P. J., & Wesely, M. L. (2000). Correcting eddy-covariance flux underestimates over a grassland. *Agricultural and Forest Meteorology*, *103*(3), 279–300. https://doi.org/10.1016/S0168-1923(00)00123-4

Wen, X.-F., Wang, H.-M., Wang, J.-L., Yu, G.-R., & Sun, X.-M. (2010). Ecosystem carbon exchanges of a subtropical evergreen coniferous plantation subjected to seasonal drought, 2003–2007. *Biogeosciences*, *7*(1), 357–369. https://doi.org/10.5194/bg-7-357-2010

Wen, X.-F., Yu, G.-R., Sun, X.-M., Li, Q.-K., Liu, Y.-F., Zhang, L.-M., Ren, C.-Y., Fu, Y.-L., & Li, Z.-Q. (2006). Soil moisture effect on the temperature dependence of ecosystem respiration in a subtropical Pinus plantation of southeastern China. *Agricultural and Forest Meteorology*, *137*(3–4), 166–175. https://doi.org/10.1016/j.agrformet.2006.02.005

Wolf, S., Eugster, W., Potvin, C., Turner, B. L., & Buchmann, N. (2011a). Carbon sequestration potential of tropical pasture compared with afforestation in Panama. *Global Change Biology*, *17*(9), 2763–2780. https://doi.org/10.1111/j.1365-2486.2011.02460.x

Wolf, S., Eugster, W., Potvin, C., Turner, B. L., & Buchmann, N. (2011b). Carbon sequestration potential of tropical pasture compared with afforestation in Panama. *Global Change Biology*, *17*(9), 2763–2780. https://doi.org/10.1111/j.1365-2486.2011.02460.x

Wolf, S., Keenan, T. F., Fisher, J. B., Baldocchi, D. D., Desai, A. R., Richardson, A. D., Scott, R. L., Law, B. E., Litvak, M. E., Brunsell, N. A., Peters, W., & Van Der Laan-Luijkx, I. T. (2016). Warm spring reduced carbon cycle impact of the 2012 US summer drought. *Proceedings of the National Academy of Sciences*, *113*(21), 5880–5885. https://doi.org/10.1073/pnas.1519620113

Xie, J., Sun, G., Chu, H.-S., Liu, J., McNulty, S. G., Noormets, A., John, R., Ouyang, Z., Zha, T., Li, H., Guan, W., & Chen, J. (2014). Long-term variability in the water budget and its controls in an oak-dominated temperate forest. *Hydrological Processes*, *28*(25), 6054–6066. https://doi.org/10.1002/hyp.10079

Yee, M. S., Pauwels, V. R. N., Daly, E., Beringer, J., Rüdiger, C., McCabe, M. F., & Walker, J. P. (2015). A comparison of optical and microwave scintillometers with eddy covariance derived surface heat fluxes. *Agricultural and Forest Meteorology*, *213*, 226–239. https://doi.org/10.1016/j.agrformet.2015.07.004

Zhang, L.-M., Yu, G.-R., Sun, X.-M., Wen, X.-F., Ren, C.-Y., Fu, Y.-L., Li, Q.-K., Li, Z.-Q., Liu, Y.-F., Guan, D.-X., & Yan, J.-H. (2006). Seasonal variations of ecosystem apparent quantum yield (α) and maximum photosynthesis rate (Pmax) of different forest ecosystems in China. *Agricultural and Forest Meteorology*, *137*(3–4), 176–187. https://doi.org/10.1016/j.agrformet.2006.02.006

Zhang, Q., Ficklin, D. L., Manzoni, S., Wang, L., Way, D., Phillips, R. P., & Novick, K. A. (2019). Response of ecosystem intrinsic water use efficiency and gross primary productivity to rising vapor pressure deficit. *Environmental Research Letters*, *14*(7), 074023. https://doi.org/10.1088/1748-9326/ab2603

Zhang, Q., Phillips, R. P., Manzoni, S., Scott, R. L., Oishi, A. C., Finzi, A., Daly, E., Vargas, R., & Novick, K. A. (2018). Changes in photosynthesis and soil moisture drive the seasonal soil respiration-temperature hysteresis relationship. *Agricultural and Forest Meteorology*, *259*, 184–195. https://doi.org/10.1016/j.agrformet.2018.05.005
